# Referrals and Management Strategies for Pediatric Obesity—*DocStyles* Survey 2017

**DOI:** 10.3389/fped.2018.00367

**Published:** 2018-12-12

**Authors:** Omoye E. Imoisili, Alyson B. Goodman, Carrie A. Dooyema, Sohyun Park, Megan Harrison, Elizabeth A. Lundeen, Heidi Blanck

**Affiliations:** ^1^Epidemic Intelligence Service, Office of Surveillance, Epidemiology, and Laboratory Services, Centers for Disease Control and Prevention, Atlanta, GA, United States; ^2^Division of Nutrition, Physical Activity, and Obesity, National Center for Chronic Disease Prevention and Health Promotion, Centers for Disease Control and Prevention, Atlanta, GA, United States

**Keywords:** pediatric obesity, obesity management, weight management programs, clinician referrals, clinician characteristics

## Abstract

**Background:** Childhood obesity care management options can be delivered in community-, clinic-, and hospital-settings. The referral practices of clinicians to these various settings have not previously been characterized beyond the local level. This study describes the management strategies and referral practices of clinicians caring for pediatric patients with obesity and associated clinician characteristics in a geographically diverse sample.

**Methods:** This cross-sectional study used data from the *DocStyles* 2017 panel-based survey of 891 clinicians who see pediatric patients. We used multivariable logistic regression to estimate associations between the demographic and practice characteristics of clinicians and types of referrals for the purposes of pediatric weight management.

**Results:** About half of surveyed clinicians (54%) referred <25% of their pediatric patients with obesity for the purposes of weight management. Only 15% referred most (≥75%) of their pediatric patients with obesity for weight management. Referral types included clinical referrals, behavioral referrals, and weight management program (WMP) referrals. Within these categories, the percentage referrals ranged from 19% for behavioral/mental health professionals to 72% for registered dieticians. Among the significant associations, female clinicians had higher odds of referral to community and clinical WMP; practices in the Northeast had higher odds of referral to subspecialists, dieticians, mental health professionals, and clinical WMP; and clinics having ≥15 well child visits per week were associated with higher odds of referral to subspecialists, mental health professionals, and health educators. Not having an affiliation with teaching hospitals and serving low-income patients were associated with lower odds of referral to mental health professionals, and community and clinical WMP. Compared to pediatricians, family practitioners, internists, and nurse practitioners had higher odds of providing referrals to mental health professionals and to health educators.

**Conclusion:** This study helps characterize the current landscape of referral practices and management strategies of clinicians who care for pediatric patients with obesity. Our data provide insight into the clinician, clinical practice, and reported patient characteristics associated with childhood obesity referral types. Understanding referral patterns and management strategies may help improve care for children with obesity and their families.

## Introduction

Childhood obesity is a serious health problem associated with both physical and psychological consequences, including hypertension, hyperlipidemia, insulin resistance, asthma, weight stigma, and bullying, among others (1–6). Approximately 18.5% of children ages 2–19 in the United States have obesity (body mass index [BMI] kg/m^2^ ≥95th percentile for age and sex) (7), and childhood obesity tracks into adulthood (8). Among children with obesity, reductions of BMI in childhood might decrease the risk of developing insulin resistance, dyslipidemia, and hypertension (9, 10), as well as have positive benefits for psychological well-being (11). To treat childhood obesity, coordinated action is needed in the places where children live, learn, and play. With 13.7 million U.S. children aged 2–19 already living with obesity (7), weight management services can help children achieve and maintain a healthy weight, and promote behavior change (12). A collective, multidisciplinary approach will likely require childhood obesity treatment and care management options delivered in community venues, clinics, and hospital-based settings, and involve different types of healthcare providers (13–16).

Clinical guidelines and recommendations exist to support childhood obesity prevention and management by healthcare providers, including the 2007 Expert Committee Recommendations Regarding the Prevention, Assessment, and Treatment of Child and Adolescent Overweight and Obesity (17), the 2017 Screening for Obesity in Children and Adolescents United States Preventive Services Task Force (USPSTF) Recommendation Statement (18), and the 2017 Endocrine Society Clinical Practice Guideline on Pediatric Obesity—Assessment, Treatment, and Prevention (19). Commonalities of these recommendations are that children be screened for obesity using BMI, and that children with obesity be referred to programs or services for the purpose of weight management. The Expert Committee recommendations promote staged prevention and treatment pathways, and the Endocrine Society also delineates clinical steps for management, whereas the USPSTF provides sufficient evidence to recommend children with obesity be referred to comprehensive behavioral interventions with moderate to high intensity, in order to promote a healthy weight status.

The implementation of these recommendations in practice likely vary, depending on factors, such as patient sociodemographics, intervention setting, payer type, and other key factors (13, 20–24). For example, referrals for pediatric weight management may include primary care providers, subspecialty providers, registered dietitians, physical or exercise therapists, health educators, behavioral counselors, mental health professionals, comprehensive, multidisciplinary pediatric weight management programs, and others. Such professionals are recognized as potential healthcare providers for children with obesity in the aforementioned recommendations. Children might also be referred to clinical subspecialists for management of comorbidities associated with obesity (25), such as those requiring management by endocrinologists or gastroenterologists due to comorbidities, such as type 2 diabetes, nonalcoholic fatty liver disease or gastroesophageal reflux disease (19, 26). Due to a lack of data beyond the local level, the current landscape of referral practices and management strategies for childhood obesity in the United States is not well understood. Exploring data that have more geographic diversity may be helpful in understanding the uptake of evidence-based practices regarding the management of childhood obesity across the nation. This paper aims to describe the management strategies and referral practices of clinicians who care for pediatric patients with obesity. We also explore clinician characteristics associated with these referrals.

## Methods

### Design

This cross-sectional study used data from the 2017 *DocStyles* survey, a web-based panel survey of U.S. clinicians designed to further understand healthcare provider practices. *DocStyles* survey is administered by Porter Novelli Public Services, a public relations and social marketing firm.

### Study Sample

Respondents were sampled from the SERMO Global Medical Panel—a global market research provider (27). From the SERMO panel, which included 51,000 primary care providers (PCPs, including internists), 12,700 Pediatricians, and 2,400 Nurse Practitioners, Porter Novelli set sample size quotas that included 1,000 primary care physicians (including family practitioners and internists), 250 pediatricians, 250 obstetrician/gynecologists, 250 nurse practitioners, 250 oncologists, 150 retail pharmacists, and 100 hospital pharmacists. When comparing physician respondents from *DocStyles* survey 2017 to physicians in the American Medical Association Physician Master File, survey respondents were more male (69.6 vs. 58.0%), slightly older (48.1 vs. 47.0 years), and practiced for a shorter duration (17.6 vs. 19.3 years). From June 8th to August 9th, 2017, responses were obtained (28). Panelists were verified via a double opt-in sign up process with telephone confirmation at their place of work. An honorarium of $23–$85 was paid to respondents for completing the survey, and was determined by the number of questions they were asked to complete.

**Figure 1:**
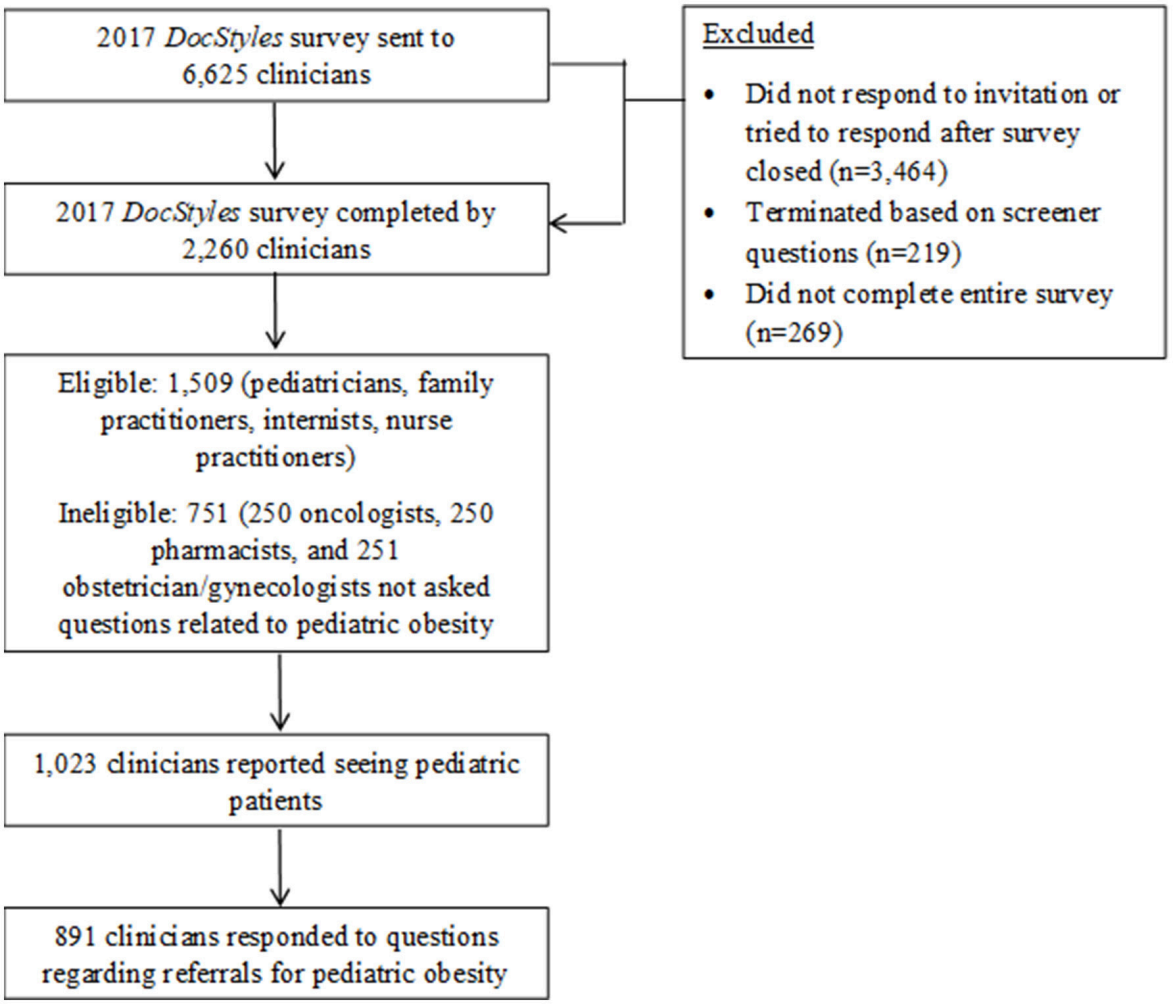
Analytic sample flow chart for *DocStyles* survey, 2017.

Eligibility criteria for *DocStyles* survey 2017 included clinicians within the United States who have actively seen patients for at least 3 years in an individual, group, or hospital practice. There were 2,260 clinicians who completed the survey (Figure 1). For this particular analysis, questions were only asked of pediatricians, internists, family practitioners and nurse practitioners (*n* = 1,509). Furthermore, use of skip patterns narrowed the respondents to clinicians who reported caring for pediatric patients (age ≤ 17 years; *n* = 1,023). The final analytic sample consisted of 891 respondents, as 132 clinicians (12.9%) had missing data on referral to weight management programs for childhood obesity. These clinicians who were excluded from this study due to missing data did not differ by demographics, but differed by specialty [higher proportion of internists (27 vs. 13%) and nurse practitioners (27 vs. 15%)] and work setting [higher proportion of inpatient clinicians (23 vs. 5%)]. The CDC licensed the results of the *DocStyles* 2017 survey post-collection from Porter Novelli. Since personal identifiers were not included in the data files, IRB approval was not needed for this project because CDC was not engaged in human subjects research.

### Outcome Measures

The primary outcomes for this analysis were clinician referrals to clinical specialists, behavioral specialists, and Weight Management Programs. The questions to clinicians were presented as follows: “The next few questions are about your referral practices for children with obesity (i.e., BMI ≥95th percentile). Among your patients aged 6–18 years with obesity, approximately what percentage do you refer to services or programs for the purpose of weight management?” Respondents provided a numeric value from 0 to 100. Subsequently, clinicians were asked: “What action(s) do you typically take for children with obesity (i.e., BMI ≥95th percentile) for the purpose of weight management?” Respondents could select all relevant options, which were not mutually exclusive. Response choices included: (1) Schedule a follow-up visit for obesity, or referral to (2) a subspecialty, such as endocrinology or gastroenterology; (3) a registered dietitian; (4) a behavioral/mental health profession; (5) a health educator/coach (6) a community-based weight management program/organization (e.g., YMCA, Weight Watchers); and (7) a clinic- or hospital-based weight management program/organization. Based on the response options, the main outcome variables for the analysis were the percentage of patients with obesity referred for services, and the types of referrals that clinicians typically made for children with obesity. Scheduling for a follow-up visit was considered a management strategy, while the other options were considered to be referrals.

### Covariates

Covariates for these analyses were grouped into clinician, clinical practice, and reported patient characteristics. Clinician characteristics included clinician age (<45 or ≥45 years), gender (male and female), and race/ethnicity (non-Hispanic white, non-Hispanic Asian, and other). Age categories were determined by prior studies (29, 30) and respondent distribution. Clinical practice characteristics were comprised of practice location Census region (Northeast, South, Midwest, West), medical specialty (family practice, internal medicine, pediatrics, nurse practitioner), primary work setting (inpatient, individual outpatient, or group outpatient), teaching hospital privileges (yes or no), and number of well-child visits per week (<5, 5–14, or ≥15 visits, reported as a continuous variable, and categories were determined by distribution of the data). Clinicians reported on two characteristics of their patient population: income and weight status. They were asked to select the income category that best described the approximate household income of the majority of their patients. These responses were subsequently grouped into three categories based on distribution of the sample, and included low-income (<$50,000), middle-income ($50,000– <$100,000), and high-income (≥$100,000). Respondents also reported what percentage of their pediatric patients had obesity; these were categorized as <10, 10– <20, 20– <40, and ≥40% based on the data distribution.

### Statistical Analysis

SAS version 9.4 (SAS Institute Inc., Cary, North Carolina) was used to perform statistical analyses. Crude associations between reported referral and personal, clinical practice, and patient characteristics were assessed by chi-squared tests; the criterion for statistical significance was *p* < 0.05. A multivariable logistic regression model estimated the adjusted odds ratios (aOR) and 95% confidence intervals (CI) for characteristics associated with referral. All covariates (i.e., clinician, clinical practice, and reported patient characteristics) were included in one model after a diagnostic assessment did not reveal significant collinearity between variables.

## Results

**Figure 2:**
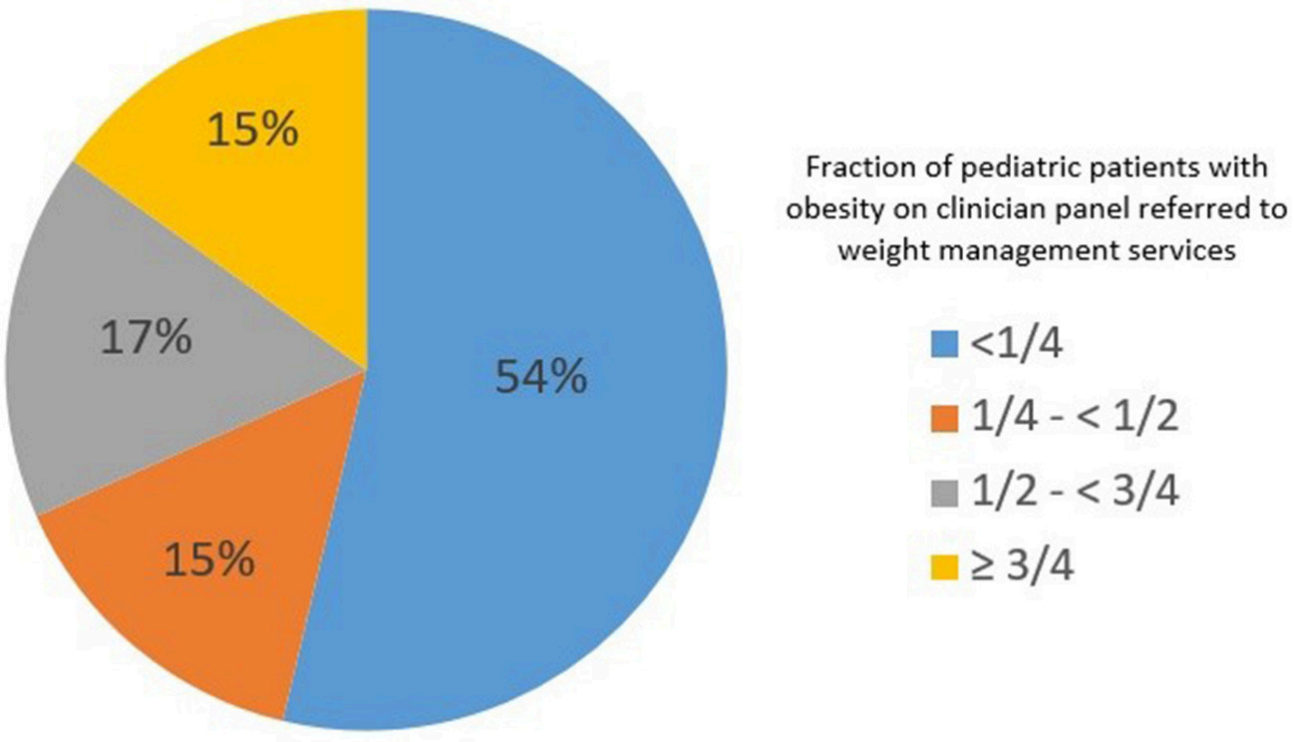
Percentage of clinicians who refer pediatric patients with obesity for weight management services, *DocStyles* survey 2017 (*N* = 891).

Table 1 shows the clinician, clinical practice, and patient characteristics of the 891 clinicians in the analytic sample. The majority of respondent clinicians were white non-Hispanic (73%) and worked in a group outpatient setting (76%). Just over half of clinicians (54%) reported referring less than a quarter of their pediatric patients with obesity for the purposes of weight management (Figure 2). Additionally, 15% reported referring 25– <50% of their pediatric patients with obesity, and 17% referred 50– <75% of their pediatric patients. Only 15% referred ≥75% of their pediatric patients with obesity for weight management.

**Table 1:** Characteristics of clinicians who see children, clinical practice, and patients, *DocStyles* survey 2017 (*N* = 891).

**Clinicians characteristics**	**All respondents *n* (%)^a^^,^^b^**
Total	891 (100)
**AGE**	
<45 years	357 (40)
≥45 years	534 (60)
**GENDER**	
Male	499 (56)
Female	392 (44)
**RACE/ETHNICITY**	
White, non-Hispanic	652 (73)
Asian, non-Hispanic	135 (15)
Other	104 (12)
**CLINICAL PRACTICE CHARACTERISTICS**	
**Census region**	
Northeast	196 (22)
South	319 (36)
Midwest	188 (21)
West	188 (21)
**Specialty**	
Family practitioner	420 (47)
Internist	113 (13)
Pediatrician	228 (26)
Nurse practitioner	130 (15)
**Work setting**	
Individual outpatient	170 (19)
Group outpatient	673 (76)
Inpatient	48 (5)
**Teaching hospital privileges**	
Yes	401 (45)
No	490 (55)
**Number of well-child visits per week**	
<5	190 (21)
5–14	324 (36)
≥15	377 (42)
**CLINICIAN REPORTED PATIENT CHARACTERISTICS**	
**Patient income**	
Low (<$50,000)	302 (34)
Middle ($50,000– < $100,000)	314 (35)
High (≥$100,000)	275 (31)
**% Pediatric patients with obesity**	
<10%	165 (19)
10– <20%	260 (29)
20– <40%	333 (37)
≥40%	133 (15)

a *Number and percentage indicates respondents who answered the question in the affirmative*.

b *Due to rounding, the sum of percentages in each category may not exactly equal 100*.

Over two-thirds of clinicians (68%) scheduled pediatric patients for follow-up visits (Table 2). The most common clinical referral reported was to a registered dietitian (72%), followed by referral to a subspecialist (27%). The most common behavioral health referral was to a health educator/coach (26%) followed by behavioral/mental health professional (19%). Finally, approximately one-third of clinicians referred their pediatric patients with obesity to weight management programs, whether community-based (35%) or hospital/clinic-based (32%) (Table 2).

**Table 2:** Characteristics of clinicians, clinical practice, and patients associated with referral practices, *DocStyles* survey 2017 (*N* = 891)^a^.

	**Clinical referral**		**Behavioral referral**			**Weight management program referral**	
	**Schedule follow-up visit, (%)**	**Subspecialty, such as endocrinology or gastroenterology, (%)**	**Registered dietitian, (%)**	**Behavioral/Mental health professional, (%)**	**Health educator/Coach, (%)**	**Community-based weight management program, (%)**	**Clinic/Hospital based weight management program, (%)**
**Total (*N* = 891)^b^**	68	27	72	19	26	35	32
**CLINICIANS CHARACTERISTICS**							
**Age**							
<45 years (*n* = 357)	67	**31**	75	19	**30**	36	33
≥45 years (*n* = 534)	68	**24**	70	18	**23**	34	32
**Gender**							
Male (n = 499)	66	25	70	19	27	**32**	**29**
Female (n = 392)	70	30	75	18	24	**39**	**37**
**Race/Ethnicity**							
White, non-Hispanic (*n* = 652)	68	28	75	19	25	35	31
Asian, non-Hispanic (*n* = 135)	64	26	70	17	25	33	32
Other (*n* = 104)	70	22	65	17	33	39	39
**CLINICAL PRACTICE CHARACTERISTICS**							
**Census region**							
Northeast (*n* = 196)	**68**	**37**	**77**	24	25	31	**39**
South (*n* = 319)	**66**	**25**	**67**	16	29	35	**28**
Midwest (*n* = 188)	**62**	**22**	**77**	18	23	36	**37**
West (*n* = 188)	**76**	**24**	**72**	20	23	38	**28**
**Specialty**							
Family practitioner (*n* = 420)	68	**20**	68	19	**26**	32	**25**
Internist (*n* = 113)	64	**27**	72	24	**42**	40	**35**
Pediatrician (*n* = 250)	69	**38**	76	14	**15**	35	**44**
Nurse practitioner (*n* = 130)	66	**29**	78	22	**32**	40	**33**
**Work setting**							
Individual outpatient (*n* = 170)	67	22	69	17	**28**	38	28
Group outpatient (*n* = 673)	68	28	73	19	**23**	34	33
Inpatient (*n* = 48)	73	31	65	17	**52**	40	35
**Teaching hospital privileges**							
Yes (*n* = 401)	70	30	73	**22**	**29**	**40**	**41**
No (*n* = 490)	66	25	71	**16**	**23**	**30**	**25**
**Number of well-child visits per week**							
< 5 (*n* = 190)	63	**18**	70	15	23	32	**28**
5–14 (*n* = 324)	69	**26**	70	19	29	32	**27**
≥15 (*n* = 377)	68	**33**	75	21	25	39	**39**
**CLINICIAN REPORTED PATIENT CHARACTERISTICS**							
**Patient income**							
Low (< $50,000) (*n* = 302)	**73**	**23**	74	18	26	35	**31**
Middle ($50,000– < $100,000) (n = 314)	**60**	**26**	72	19	27	37	**38**
High (≥$100,000) (*n* = 275)	**71**	**33**	70	20	24	32	**27**
**% Pediatric patients with obesity**							
<10%	71	24	70	21	**31**	38	33
10– < 20%	65	29	76	17	**22**	32	32
20– < 40%	66	29	73	19	**23**	33	35
≥40%	72	23	65	20	**33**	42	28

a *Bold font indicates statistical significance based on chi-square test for report of referral to each option (selected or not selected), P < 0.05*.

b *Number and percentage indicates respondents who answered the question in the affirmative*.

When we examined clinician characteristics, odds of clinical referrals to subspecialists were lower among clinicians ≥45 years of age (aOR = 0.7, 95% CI = 0.5–1.0) compared to clinicians < 45 years (Table 3). Female clinicians had higher odds of referral to both community- and clinic/hospital-based weight management programs (aOR = 1.4, 95% CI = 1.0–2.0, and aOR = 1.5, 95% CI = 1.1–2.1, respectively).

**Table 3:** Adjusted Odds Ratios^a^ of pediatric obesity referral practices by characteristics of clinicians, clinical practice, and patients, *DocStyles* survey 2017 (*N* = 891)^b^.

	**Clinical referral**			**Behavioral referral**		**Weight management program referral**	
	**Schedule follow-up visit, aOR (95% CI)^c^**	**Subspecialty, such as endocrinology or gastroenterology, aOR (95% CI)**	**Registered dietitian, aOR (95% CI)**	**Behavioral/Mental health professional, aOR (95% CI)**	**Health educator/Coach, aOR (95% CI)**	**Community-based weight management program, aOR (95% CI)**	**Clinic/Hospital based weight management program, aOR (95% CI)**
**CLINICIANS CHARACTERISTICS**							
**Age**							
<45 years	1.0	1.0	1.0	1.0	1.0	1.0	1.0
≥45 years	1.2 (0.8–1.6)	**0.7 (0.5–1.0)**	0.8 (0.6–1.1)	1.0 (0.7–1.5)	0.8 (0.6–1.1)	1.0 (0.7–1.3)	1.0 (0.7–1.3)
**Gender**							
Male	1.0	1.0	1.0	1.0	1.0	1.0	1.0
Female	1.3 (0.9–1.8)	1.2 (0.9–1.7)	1.2 (0.8–1.6)	0.9 (0.6–1.4)	0.7 (0.5–1.1)	**1.4 (1.1–2.0)**	**1.5 (1.1–2.1)**
**Race/Ethnicity**							
White, non-Hispanic	1.0	1.0	1.0	1.0	1.0	1.0	1.0
Asian, non-Hispanic	0.8 (0.6–1.3)	0.8 (0.5–1.2)	0.9 (0.6–1.3)	0.8 (0.5–1.3)	0.9 (0.6–1.4)	0.8 (0.5–1.3)	1.0 (0.7–1.6)
Other	1.0 (0.6–1.6)	0.7 (0.4–1.1)	0.7 (0.4–1.1)	0.8 (0.5–1.5)	1.3 (0.8–2.1)	1.0 (0.7–1.6)	1.4 (0.9–2.2)
**CLINICAL PRACTICE CHARACTERISTICS**							
**Census region**							
Northeast	1.1 (0.7–1.6)	**1.5 (1.0–2.2)**	**1.6 (1.0–2.4)**	**1.6 (1.0–2.6)**	0.8 (0.5–1.2)	0.8 (0.5–1.1)	**1.5 (1.0–2.2)**
South	1.0	1.0	1.0	1.0	1.0	1.0	1.0
Midwest	0.9 (0.6–1.3)	0.8 (0.5–1.3)	**1.6 (1.1–2.5)**	1.1 (0.6–1.7)	0.7 (0.4–1.1)	1.0 (0.7–1.5)	**1.6 (1.1–2.4)**
West	**1.8 (1.2–2.7)**	0.9 (0.6–1.3)	1.3 (0.9–1.9)	1.4 (0.8–2.2)	0.8 (0.5–1.2)	1.2 (0.8–1.8)	1.0 (0.7–1.5)
**Specialty**							
Family practitioner	1.2 (0.8–1.8)	**0.5 (0.3–0.8)**	0.7 (0.5–1.1)	**2.4 (1.4–4.0)**	**2.8 (1.7–4.5)**	1.2 (0.8–1.9)	**0.5 (0.3–0.8)**
Internist	0.9 (0.5–1.7)	0.8 (0.5–1.5)	1.0 (0.6–1.8)	**3.5 (1.8–6.8)**	**5.1 (2.7–9.4)**	1.7 (1.0–2.9)	0.8 (0.4–1.3)
Pediatrician	1.0	1.0	1.0	1.0	1.0	1.0	1.0
Nurse practitioner	0.9 (0.5–1.5)	0.7 (0.4–1.3)	1.2 (0.6–2.1)	**3.0 (1.6–5.8)**	**4.2 (2.3–7.8)**	1.4 (0.8–2.4)	0.7 (0.4–1.2)
**Work setting**							
Individual outpatient	1.0 (0.7–1.5)	0.8 (0.5–1.3)	0.9 (0.6–1.4)	0.8 (0.5–1.3)	1.1 (0.7–1.7)	1.2 (0.8–1.8)	0.9 (0.6–1.4)
Group outpatient	1.0	1.0	1.0	1.0	1.0	1.0	1.0
Inpatient	1.3 (0.7–2.6)	1.0 (0.5–2.0)	0.6 (0.3–1.2)	0.6 (0.3–1.5)	**2.7 (1.4–5.3)**	1.1 (0.6–2.0)	0.9 (0.5–1.7)
**Teaching hospital privileges**							
Yes	1.0	1.0	1.0	1.0	1.0	1.0	1.0
No	0.8 (0.6–1.1)	1.0 (0.7–1.4)	1.0 (0.7–1.4)	**0.7 (0.5–1.0)**	0.8 (0.5–1.1)	**0.6 (0.4–0.8)**	**0.5 (0.4–0.7)**
**Number of well-child visits per week**							
<5	1.0	1.0	1.0	1.0	1.0	1.0	1.0
5–14	1.3 (0.9–2.0)	**1.7 (1.0–2.6)**	1.0 (0.7–1.5)	1.3 (0.8–2.1)	1.4 (0.9–2.2)	1.0 (0.7–1.5)	0.9 (0.6–1.3)
≥15	1.3 (0.8–2.0)	**1.7 (1.0–2.9)**	1.2 (0.7–1.8)	**2.2 (1.3–3.7)**	**1.9 (1.2–3.1)**	1.5 (1.0–2.3)	1.1 (0.7–1.7)
**CLINICIAN REPORTED PATIENT CHARACTERISTICS**							
**Patient income**							
Low (< $50,000)	1.1 (0.8–1.6)	**0.6 (0.4–0.9)**	1.3 (0.9–1.9)	0.9 (0.6–1.4)	1.1 (0.8–1.7)	1.1 (0.80–1.63)	1.4 (0.9–2.00)
Middle ($50,000– ≤ $100,000)	**0.6 (0.5–0.9)**	0.8 (0.5–1.1)	1.1 (0.8–1.6)	0.9 (0.6–1.4)	1.2 (0.8–1.8)	1.3 (0.9–1.8)	**1.9 (1.3–2.7)**
High (≥$100,000)	1.0	1.0	1.0	1.0	1.0	1.0	1.0
**% Pediatric patients with obesity**							
<10%	1.0	1.0	1.0	1.0	1.0	1.0	1.0
10– < 20%	0.8 (0.5–1.2)	1.3 (0.8–2.0)	1.4 (0.9–2.1)	0.7 (0.4–1.2)	0.7 (0.4–1.1)	0.8 (0.5–1.2)	0.8 (0.5–1.3)
20– < 40%	0.8 (0.5–1.2)	1.2 (0.8–1.9)	1.1 (0.7–1.7)	0.8 (0.5–1.4)	0.7 (0.5–1.1)	0.8 (0.5–1.2)	0.9 (0.6–1.4)
≥40%	1.0 (0.6–1.7)	0.9 (0.5–1.5)	0.7 (0.4–1.2)	0.9 (0.5–1.5)	1.0 (0.6–1.7)	1.0 (0.6–1.7)	0.7 (0.4–1.1)

a *All provider personal, medical practice, and patient characteristics were included in one model for each referral option*.

b *Bold font indicates statistical significance as 95% confidence interval does not include 1.0*.

c *Adjusted odds ratio (95% confidence interval)*.

Among the clinical practice characteristics, we found significant differences in referral practices by Census region and clinical subspecialty. Clinicians based in the West had higher adjusted odds of scheduling a follow-up visit vs. clinicians in the South (aOR = 1.8, 95% CI = 1.2–2.7). Odds of a clinical referral to a dietitian were higher in the Northeast and the Midwest vs. the South (aOR = 1.6, 95% CI = 1.0–2.4; aOR = 1.6, 95% CI = 1.1–2.5, respectively). Odds of referrals to a behavioral/mental health professional were higher in the Northeast compared to the South (aOR = 1.6; 95% CI = 1.0–2.6). Odds of referral to a hospital or clinic based weight management program were higher in in the Northeast and the Midwest vs. the South (aOR = 1.5, 95% CI = 1.0–2.2 and aOR = 1.6, 95% CI = 1.1–2.4, respectively). Clinical practice specialty was also associated with clinical referrals; family practitioners were less likely to refer to subspecialists compared to pediatricians (aOR = 0.5, 95% CI = 0.3–0.8). For behavioral referrals, all other specialties (family practitioners, internists, and nurse practitioners) had significantly higher odds of referrals to mental health professionals (aOR range: 2.4–3.5) or health educators (aOR range: 2.8–5.1) compared to pediatricians. Clinicians who primarily work in inpatient settings had higher odds of referral to health educators/coaches (aOR = 2.7, 95% CI = 1.4–5.3) compared to clinicians in group outpatient settings. Clinicians without teaching hospital privileges had lower odds of referring to a mental health professional (aOR = 0.7, 95% CI = 0.5–1.00) vs. clinicians with privileges. Finally, clinicians in practices that perform ≥5 well child visits per week had significantly higher odds of referral to subspecialists compared to those in practices that perform less than five per week (aOR: 1.7). Additionally, clinicians in practices that perform ≥15 well child visits per week had approximately twice the odds of a behavioral health referral (aOR range: 1.9–2.2) compared to those in practices that performed less than five per week.

Referral patterns also differed by clinician reported patient characteristics. When compared with clinicians serving high-income patient populations, clinicians serving low-income patient populations had lower odds of referral to a subspecialist (aOR = 0.6, 95% CI = 0.4–0.9). In addition, clinicians serving patients with middle-income populations had lower odds of scheduling a follow-up visit (aOR = 0.6; 95% CI = 0.5–0.9), but had higher odds of referral to a clinic/hospital-based weight management program (aOR = 1.9; 95% CI = 1.3–2.7). Odds of specific types of referrals did not differ based on the estimated percentage of children in the clinician's practice who had obesity.

## Discussion

Overall, about two-thirds of clinicians in this study reported scheduling patients for follow-up appointments as a management strategy for pediatric obesity. Approximately half of respondents reported referring less than one-quarter of their pediatric patients with obesity to services or programs for the purposes of weight management, and only 15% reported referring most (≥75%) of their pediatric patients with obesity.

The most common referral made among survey respondents was to registered dietitians, with almost three-quarters of clinicians reporting referral of children with obesity to this profession. Dietitians are specifically trained in nutrition and can be key members of multidisciplinary obesity management teams. A recent review found that for adults with type 2 diabetes, individual nutrition therapy conducted by dietitians resulted in better health outcomes, including a lower BMI, than care by other providers, such as physicians and nurses (31).

Approximately one-quarter of respondents reported referral to clinical subspecialists; similarly about one-quarter of surveyed clinicians also reported referral to health educators/coaches. However, we found that less than one in five reported referring pediatric patients with obesity to behavioral/mental health professionals. About one-third of clinicians referred pediatric patients with obesity to either a community-based or a clinic/hospital-based weight management programs. All of the aforementioned referral practices are potential referral options presented within the Expert Committee and USPSTF recommendations for childhood obesity. This study is the first to examine the frequency with which these referrals are made using data beyond one geographic location within the country. We also found that these referral practices differed based on clinician, clinical practice, and reported patient characteristics.

### Clinician Characteristics

Older clinicians had lower odds of subspecialty referral compared to younger physicians in our study. A previous study of referral practices among primary care physicians found that years in practice, which likely correlates with physician age, was associated with lower subspecialist referral (32). Female clinicians in this study had higher odds of referral to both types of weight management programs. Women have been shown to be more likely to engage in preventive care, and more likely to make referrals to weight loss programs for adult patients with obesity to weight loss programs (33, 34). Our results are consistent with these findings, but demonstrated this association within a pediatric patient population with obesity.

### Clinical Practice Characteristics

Respondents in the Northeast had higher odds of referrals to subspecialties, registered dietitians, behavioral/mental health professionals, and clinic/hospital-based weight management programs vs. those in the South. The Northeast region generally has a higher concentration of urban areas compared to other regions in the United States (35). Therefore, within this region resources for patients with obesity might be more attainable, with fewer geographic barriers, such as distance prohibiting access to weight management programs (36). Professionals, such as dietitians are more concentrated in the Northeast and Midwest (37). These factors could possibly contribute to the higher odds of referral to dietitians and clinic based weight management programs in the Midwest vs. the South.

Clinician specialty was also associated with some referrals, with all surveyed non-pediatric specialties having higher odds of behavioral referrals for childhood obesity, compared to pediatricians. Pediatricians are trained specifically to care for children and might have been taught about pediatric weight management while in training (38, 39). While self-efficacy for weight management holds value, evidence suggests non-traditional healthcare providers, such as health coaches (40) might be beneficial in pediatric weight management. In addition, inter-professional collaborations (i.e., between dietitians, exercise therapists, and others) are beneficial for childhood obesity management (17, 23). Family practitioners had lower odds of referral to clinical subspecialists, and to clinic/hospital-based weight management programs, compared to pediatricians. This is consistent with a prior study that also documented significant differences between the approaches of family practitioners and pediatricians in the treatment of children, with family physicians in that study having significantly lower odds of referral of pediatric patients for further evaluation and management for weight related care (41).

Work setting and teaching hospital privileges were also associated with respondents' likelihood of behavioral referrals. Clinicians who practice inpatient had higher odds of referring to a health educator/coach in our study. These clinicians might be able to take advantage of inpatient health educators during patient admissions (42). Respondents without teaching hospital privileges had lower odds of referral to a behavioral/mental health professional and to clinic/hospital based weight management programs in the present study. It is possible that privileges with a teaching hospital could mean belonging to a referral network that facilitates patient access to such resources. For instance, a higher percentage of teaching hospitals offer psychiatric outpatient services compared to non-teaching hospitals (43).

In our study, the number of well-child visits per week per practice was associated with increased odds of referrals to clinical subspecialties and behavioral referrals. A greater number of well child visits per week might be indicative of a larger or more pediatric focused practice. In a study among primarily family practitioners, clinicians in solo or small group practice were less likely to make referrals compared to physicians in larger practices (32). Having larger patient volumes might mean less time per patient (44), and potentially more referrals.

### Reported Patient Characteristics

In the present study, clinicians working with middle-income patients were less likely to schedule patients for a follow-up visit; however, they also had higher odds of referring their patients externally to clinic/hospital-based weight management programs. A previous study demonstrated that attending a pediatric weight management program after referral is associated with socioeconomic status and insurance status (45). However, data have not formally shown whether perceived likelihood of patient attendance affects clinician odds of referral. In addition, clinicians who reported caring for low-income patients had a lower odds of referrals to clinical subspecialists. It might be more difficult for patients with lower incomes to find subspecialists who will take their insurance, such as Medicaid (46–48).

### Strengths and Limitations

This study is subject to several limitations. First, the *DocStyles* survey is a panel survey based on quota sampling, resulting in a sample that is not necessarily representative of the population, and thus the findings might not be generalizable to clinicians nationwide. In addition, survey respondents may differ compared to those who did not participate in the survey. However, this study uses a sample with more geographic diversity than previous samples rather than a limited local sample. Second, *DocStyles* data are based on the report of clinicians both about their personal practices and perceptions about their patient population; no objective measures were obtained. Thus, their responses are subject to reporting biases. Nevertheless, querying clinicians directly can provide insight into what influences their referral practices. Finally, the possibility exists that there are factors that confound or modify the association between clinician, clinical practice, or clinician reported patient characteristics and referral type that might not have been accounted for; for example, location in an urban or rural environment. Despite these limitations, this study adds valuable information to the literature by describing current practice and characteristics that may influence childhood obesity referral practices of clinicians in the United States.

## Conclusion

In this study, clinicians caring for children with obesity referred patients to a wide range of providers and services for the purposes of weight management; although half of clinicians referred less than one-quarter of their pediatric patients with obesity for these interventions. Most respondents referred pediatric patients with obesity to dietitians, and the majority scheduled follow-up appointments, which is a recommended practice. Clinicians also referred to clinic-based weight management programs, clinical subspecialists, health educators, and behavioral/mental health professionals with varying frequencies, ranging from 19% for behavioral/mental health professionals to 72% for registered dieticians. Referrals were generally consistent with recommendations from the AAP or USPSTF to address the complex nature of childhood obesity management. Our findings contribute to understanding the current landscape of referral practices and management strategies of clinicians who care for pediatric patients with obesity. Our data also provide insight into the clinician, clinical practice, and reported patient characteristics associated with childhood obesity referral types. Understanding referral patterns and management strategies can help inform strategies to improve the uptake of current recommended care.

## Author Contributions

OI, AG, CD, SP, MH, EL, and HB wrote and contributed in the preparation of this manuscript. AG, CD, MH, and HB formulated research questions explored in this study, and contributed subject matter expertise. OI, SP, and EL engaged in statistical analysis of the survey data.

### Conflict of Interest Statement

The authors declare that the research was conducted in the absence of any commercial or financial relationships that could be construed as a potential conflict of interest.
